# Typhoid Fever in a Horse1Presented at the Fourteenth Annual Meeting of the Iowa State Veterinary Medical Association, Des Moines, Iowa, February 11 and 12, 1902.

**Published:** 1902-04

**Authors:** L. U. Shipley

**Affiliations:** Sheldon, Iowa


### TYPHOID FEVER IN A HORSE.[1]

By L. U. Shipley, D.V.S.,
SHELDON, IOWA.

The subject of this report was a bay gelding, six years old' weighing about 1100 pounds, that was bought by a dealer and put on feed to condition him for the market. About a week or ten days later our attention was called to the case. The groom stated that he did not eat well. After a casual examination we concluded that it was a case of indigestion, and treated him accordingly. However, his condition did not improve. By this time he had begun to show emaciation, eating sparingly of hay and grain at times, and at other times refusing grain entirely and drinking but little water. He was observed to yawn or gape and to grind the teeth and assume a position when standing similar to that of a horse about to urinate, seemingly desiring to tense the abdominal muscles. He had a tucked-up appearance of the flanks, and was also restless when lying down. The feces were of natural color but soft, and resembled in consistency those of a cow. Loud borborygmi were constantly present. The temperature ranged about 103° F., the pulse about 72, but otherwise normal in character; respiration was normal; the visible mucous membrane presented some small petechial spots. Pressure over the abdominal region seemed to cause no perceptible pain. The hair was sleek and glossy throughout the course of the disease. The case remained about the same except for a progressive emaciation, presenting the foregoing symptoms, with more or less intensity, for some eight weeks, when the symptoms became more alarming, and he died the following night.

The next forenoon we made a post-mortem examination and found considerable wine-colored fluid in the abdominal cavity. The peritoneum presented a thickened, softened condition, showing peritonitis to have been the immediate cause of death. Upon removing the small intestines we found the mucous layer much tumefied, as though having been affected by a catarrhal inflammation for some time. Peyer's and other lymph glands presented different stages of ulceration, with one or more distinct perforations. These ulcers were distributed throughout the length of the

[1] Presented at the Fourteenth Annual Meeting of the Iowa State Veterinary Medical Association, Des Moines, Iowa, February 11 and 12, 1902.

small intestines, and varied in size from that of the end of a lead-pencil to one inch in diameter, many of them presenting a yellowish-brown scab or slough not yet thrown off. All other digestive organs were normal in appearance.

After having searched all the veterinary text-books at our command we sought some information upon this interesting case from works upon human pathology, and in their treatises on typhoid fever we found the following summary: "An endemic, infectious fever, associated with constant lesions of the lymph follicles of the intestines; first, hyperplasia, followed by ulceration of the solitary Peyer's and other lymph glands, due to the bacillus of Eberth."

The symptoms of the foregoing case—first, the continual presence of fever; second, the peculiar looseness of the bowels; third, the ulceration, perforation, and consequent peritonitis—are all characteristics of typhoid fever in man; consequently, the application of the title of this report. That the lesions were due to the bacillus of Eberth, as in man, we have no proof, having made no bacteriological search.

### Discussion.

Dr. Repp said that inasmuch as eminent investigators are unanimous in the opinion that typhoid fever as we know it in the human being does not occur in the domestic animals, he could not adopt the opinion of the author of this report that this was a case of typhoid fever.

